# Quality of Ventilations during Infant Resuscitation: A Simulation Study Comparing Endotracheal Tube with Face Mask

**DOI:** 10.3390/children9111757

**Published:** 2022-11-16

**Authors:** Myriam Santos-Folgar, Paula Lafuente-Filgueira, Martín Otero-Agra, Felipe Fernández-Méndez, Roberto Barcala-Furelos, Javier Trastoy-Quintela, Silvia Aranda-García, María Fernández-Méndez, Antonio Rodríguez-Núñez

**Affiliations:** 1 REMOSS Research Group, Faculty of Education and Sport Sciences, University of Vigo, 36005 Pontevedra, Spain; 2 School of Nursing, University of Vigo, 36001 Pontevedra, Spain; 3 Department of Obstetrics, Complexo Hospitalario of Pontevedra, Sergas, 36002 Pontevedra, Spain; 4 Complexo Hospitalario of Pontevedra, Sergas, 36002 Pontevedra, Spain; 5 Life Support and Simulation, Institute of Research of Santiago, CLINURSID Research Group, University of Santiago de Compostela, Santiago de Compostela, 15706 A Coruña, Spain; 6 Faculty of Education and Sports Sciences, University of Vigo, 36005 Pontevedra, Spain; 7 Paediatric Critical, Intermediate and Palliative Care Section, Hospital Clínico Universitario de Santiago de Compostela, 15706 A Coruña, Spain; 8 GRAFIS Research Group, Institut Nacional d’Educació Física de Catalunya (INEFC), Universitat de Barcelona, 08007 Barcelona, Spain

**Keywords:** infant, cardiopulmonary resuscitation (CPR), ventilation, quality, pressure, training

## Abstract

Background: There are few studies that analyze ventilation volume and pressure during CPR carried out on infants. The aim of this study was to evaluate the quality of the ventilations administered using a self-inflating bag with an endotracheal tube and a face mask in manikins. Methods: a quasi-experimental simulation study with a randomized case crossover design [endotracheal tube (ET) vs. face mask (FM)] was performed. Sixty participants who were previously trained nursing students participated in the study. The estimated air volumes breathed, and the pressure generated during each ventilation were assessed and the quality of the chest compressions was recorded. Results: the ET test presented a higher percentage of ventilations that reached the lungs (100% vs. 86%; *p* < 0.001), with adequate volume (60% vs. 28%; *p* < 0.001) in comparison to FM. Both tests presented peak pressures generated in the airway greater than 30 cm H_2_O (ET: 22% vs. FM: 31%; *p* = 0.03). Conclusions: performing quality CPR ventilations on an infant model is not an easy skill for trained nursing students. Both tests presented a significant incidence of excessive peak pressure during ventilations. Specific training, focused on quality of ventilations guided by a manometer attached to the self-inflating bag, must be considered in life support training for pediatric providers.

## 1. Introduction

Infant cardiac arrest is often caused by asphyxia and, therefore, the quality of the ventilation is essential during cardiopulmonary resuscitation (CPR) [1]. Hypoxia, hypocapnia and hypercapnia (a consequence of inadequate oxygenation and/or ventilation during CPR) have been found to be poor prognostic factors in cardiac arrest both in children and adults [2,3,4,5]. Despite this, very few studies have been conducted so far, which actually assess the ability of rescuers to ventilate victims, whether they are real [6] or simulated models [7,8].

For several years now, major efforts have been made to measure the quality of chest compressions (CC) during CPR, taking into account their prognostic value and usefulness during the practical training of various groups [9,10,11]. Furthermore, devices have been designed, which enable this procedure quality to be monitored in real time [12,13,14]. Currently, the measurement of CC quality is an essential tool when learning CPR since it allows the students to receive immediate feedback on the effectiveness of the maneuvers performed [15,16,17].

Recently, some of these monitoring devices have also been able to give information on ventilation quality, although this function is limited to the detection of whether or not air actually enters the airway along with an estimate of the volume of each insufflation [18]. However, effective ventilation, in addition to an adequate volume of air, must also have an adequate pressure, and the usual training devices do not assess this parameter. We have carried out the present study taking the hypothesis that future health professionals, previously trained and capable of performing good quality CC in an infant model, would perform manual ventilations of insufficient quality during CPR. Our objective was to assess insufflation volumes and peak pressures during CPR in a simulated infant, comparing tracheal tube and face mask techniques.

## 2. Materials and Methods

### 2.1. Study Design

A quasi-experimental, randomized crossover and simulation study was designed in order to evaluate the quality of each ventilation during a CPR simulation using a self-inflating bag connected to an endotracheal tube (ET) and a face mask (FM), performed on an infant-sized manikin.

### 2.2. Sample

The sample size was based on an assumed minimum of an effect size (ES) of 0.5, an error probability of 0.05, and a statistical power of 0.95. These assumptions provided a sample size of 57 study participants computed by G*Power 3.1.9.2 software (Heinrich-Heine-Universität, Düsseldorf, Germany). The final sample consisted of 60 students from Pontevedra Nursing School (University of Vigo). The inclusion criteria were as follows: (i) having previously trained in pediatric CPR in accordance with the 2021 recommendations of the European Resuscitation Council [1]; (ii) obtaining a CC quality rate of at least 80% in a practical pre-test using a manikin; and (iii) performing hospital clinical practices at the time of the study.

### 2.3. Ethics

The research adhered to the ethical principles of the Declaration of Helsinki and was approved by the Ethics Committee of the Faculty of Education and Sports Science of the University of Vigo (code: 03-1812-17; approved on 2 March 2018). The participants signed the corresponding informed consent, and their personal data was handled anonymously.

### 2.4. Development of the Test and Data Collection

**Figure 1:**
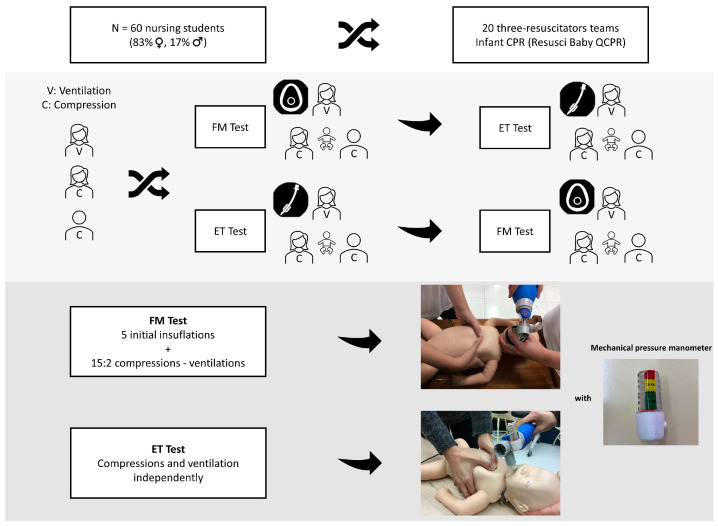
Study design flow-chart. (FM: face mask; ET: endotracheal tube).

Teams of three rescuers were randomly formed and they carried out two 5-min CPR simulations with a sequence that was also random in which the ventilation method varied (Figure 1):

- ET test: Ventilation through the endotracheal tube, performing CC and ventilation independently [1].
- FM test: Ventilation through the face mask, performing cycles of 15 CC and 2 ventilations [1].

In each resuscitation team, the ventilation was always performed by just one of the participants, positioned at the head of the manikin. Meanwhile the other team members performed the CC, switching every two minutes. CC parameters were studied to assess the influence of ventilation technique on these variables. The participants received no information or feedback on the quality of their maneuvers or the manometer readings at any time during the study. 

The following variables were recorded:

(a) Ventilation: Frequency (breaths per minute), mean ventilation volume (in mL and mL/kg), percentage of ventilations that reached the lungs, the percentage of ventilations with achieved volume labelled as insufficient (less than 35 mL or 6 mL/kg), optimal and excessive (greater than 55 mL or 10 mL/kg) and the percentage of ventilations in which peak pressure generated was greater than 30 cm H_2_O. The volume of each ventilation was recorded continuously, while the pressure was grouped into five ranges: <10 cm H_2_O, (10–19) cm H_2_O, (20–29) cm H_2_O, (30–39) cm H_2_O and (40–60) cm H_2_O.
(b) Chest compressions: Mean compression rate (compressions per minute), mean compression depth (mm), percentage of CC with correct hand position and percentage of CC with adequate release.

### 2.5. Simulation Material and Measuring Instruments

The CPR simulation was performed on the Laerdal Resusci Baby QCPR manikin (Stavanger, Norway) equipped with Laerdal ResusciAnne Wireless SkillReporter^®^ (Stavanger, Norway, v.2.0.0.14). This device detects and records the ventilations performed and the estimated volume of each ventilation in mL, in addition to the quality parameters for CC (depth in mm, rate in compressions/minute, hand positioning, CC release, compression times and “no flow”). The manufacturer has not specified the approximate age of the manikin used for simulation, so the World Health Organization (WHO) growth tables [19] were used to identify its anthropometric parameters. According to that, the simulator would correspond to a 3-month-old baby weighing approximately 5.5 kg. The target quality parameters were established as follows: ventilation tidal volumes from 6–10 mL/kg (35–55 mL) [20,21], CC depth from 36–44 mm (which is between 1/3 and 40% of the thoracic diameter) and CC rate from 100–120/min [1].

Ventilations were performed using an Ambu^®^ Mark IV pediatric sized self-inflating bag (500 mL). This resuscitator is equipped with a pressure relief valve that can be activated at insufflation pressures above 40 cm H_2_O (depending on the manufacturer) [22] and that was kept closed during the simulation tests. It also has a connection for an Ambu^®^ disposable mechanical pressure manometer prescribed in cm H_2_O (Ambu^®^, Ballerup, Copenhagen, Denmark) which was used to measure the peak pressures generated with each ventilation attempt.

Due to the fact that this manikin and its breathing quality hardware/software does not measure or estimate the peak pressures delivered while ventilating the manikin, and the physical characteristics of the plastic lung and chest cage are not comparable with real lungs and chests, we have checked the correlation between volumes and peak pressures by means of a series of tests with a mechanical ventilator. These tests were performed prior to the start of the participants’ tests to check the reliability of the devices used to measure volume and pressure, using a ventilator that monitors these parameters. (Hamilton T1, Bonaduz, Switzerland). Firstly, we delivered a series of ventilations programmed at successive tidal volumes, from 24 to 60 mL (at 6 mL increase intervals); the mean peak pressure obtained after 10 mechanical ventilations was recorded and plotted at each volume point. Secondly, we performed the reverse test, this time selecting mechanical peak pressures from 5 to 45 cm H_2_O, at 5 cm H_2_O intervals and measuring the mean tidal volumes achieved. 

Since the manikin used does not have an anatomical airway, an endotracheal tube was inserted into the manikin’s airway (i.e., a tube connecting the mouth to the baby’s simulated lung), removing the jawbone from the manikin. The tube used was a standard Mallinckrodt^®^ No. 3.0 model (Mallinckrodt^®^, Blanchardstown, Dublin, Ireland) with a balloon (consisting of a 3.0 mm internal diameter and a 4.3 mm external diameter). It was introduced far enough away so that it would not get obstructed by the manikin’s neck movements.

In the FM tests, a round Ambu^®^ face mask was used (Ambu^®^, Ballerup, Copenhagen, Denmark) of 0A size with a 37.7 mm internal diameter and a 73.1 mm external diameter.

For the subsequent, detailed recording and analysis of the data, the events were recorded using two video cameras oriented to the pressure manometer and allowing the manikin’s chest to be viewed at the same time. The brands used were GoPro^®^ (GoPro Inc. ^®^, San Mateo, CA, USA) and Sony^®^ Alpha a6000 (Sony^®^, Tokyo, Japan).

### 2.6. Statistical Analysis

Pearson’s correlation coefficient was calculated in order to analyze the correlation between volumes and pressures (and vice versa) during the preliminary test performed with the mechanical ventilator and the manikin. In the case of dichotomous variables, a descriptive analysis was performed using relative and absolute frequencies. In the case of continuous variables, a descriptive analysis was carried out using central tendency measures (median) and dispersion [interquartile range (IQR)]. In order to make the comparisons, first of all the distributions were checked for normality with the Kolmogorov-Smirnov test. After that, a Student t-test for related samples was used and a Wilcoxon range-sum test with a confidence level of *p* = 0.05 according to the normality of the variables distribution. The statistical analysis was performed using IBM SPSS Statistics version 20 software for Windows (SPSS, Inc., Armonk, NY, USA).

## 3. Results

60 nursing students (50 women and 10 men) participated in this study. In relation to demographic variables, they had a median age of 21 years (IQR: 20–24), a median weight of 62 kg (IQR: 55–72) and a median height of 165 cm (IQR: 160–170).

### 3.1. Pre-Test Ventilation Pressure-Volume

Good correlations between the mechanical tidal volumes set and generated pressures (r = 0.99 for pressure-volume and r = 0.95 for volume-pressure, *p* < 0.001) were observed. 

### 3.2. CPR Test

Overall, 600 minutes of CPR were analyzed, which included 54.822 CC and 6.671 ventilations from a total of 60 participants (20 resuscitation teams) using both ventilation techniques.

There were statistically significant differences between ET and FM in all ventilation variables except for the frequency (Table 1). In comparison to the FM test, the ET test presented a higher percentage of ventilations that reached the lungs (ET: 100%, IQR 100–100; FM: 98%, IQR 81–100; *p* < 0.001) and with adequate volume (ET: 68%, IQR 35–88; FM: 22%, IQR 8–41; *p* < 0.001). On the other hand, a lower percentage of ventilations with insufficient (ET: 0%, IQR 0–1; FM: 5%, IQR 2–10; *p* < 0.001) and with excessive volume (ET: 29%, IQR 11–62; FM: 53%, IQR 29–73; *p* = 0.04) were measured in ET in comparison with the FM test. Mean tidal volumes were also lower in the ET than in the FM test (ET: 50 ml, IQR 46–56; FM: 57 ml, IQR 51–69; *p* = 0.02). The peak pressures generated in the airway were greater than 30 cm H_2_O in a median of 0% of the ventilations carried out with the ET (IQR 0–41), while this was 18% (IQR 0–56) in the case of the FM (*p* = 0.03). 

**Figure 2:**
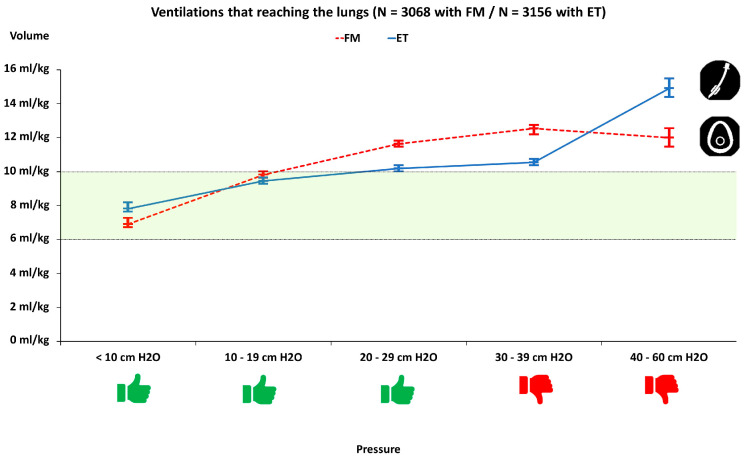
Relationship between pressures generated in the manikin’s airway and estimated volumes. FM: Face mask; ET: Endotracheal tube. Categories with a pressure of less than 30 cm H_2_O were considered safe.

The relationship between the generated pressure with all the effective ventilations and the delivered tidal volumes is shown in Figure 2. In both tests, an increase in pressure related to the increase in volume was assessed. Ventilations with pressures close to and greater than 30 cm H_2_O tended to be those that exceeded the volume recommendations in the infant victim.

**Figure 3:**
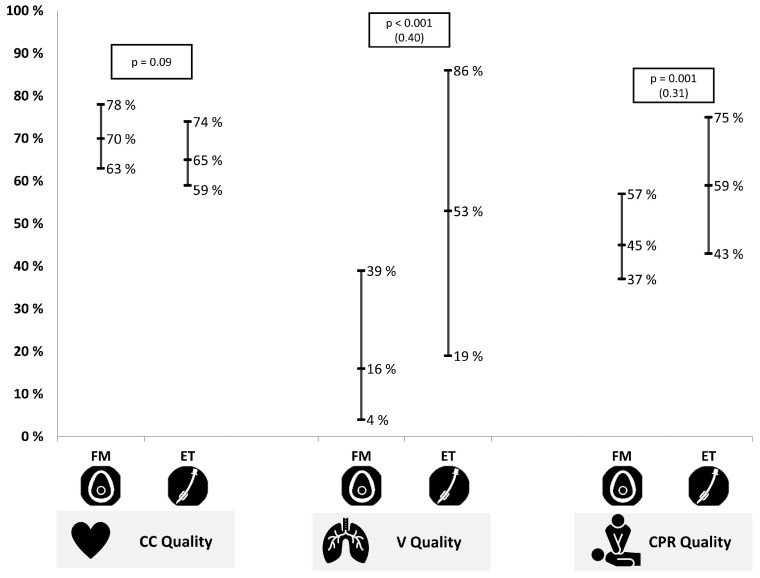
Chest compression, (CC), ventilation (V) and cardiopulmonary resuscitation (CPR) quality for the face mask (FM) and the endotracheal tube (ET) test of the ventilation quality was carried out with adequate volume and pressure. Values were expressed as the median and interquartile range. The effect size is in brackets.

CC quality was similar in both tests, except for adequate release, which was better for the FM (median 93%, IQR 74–99) than for the ET test (median 81%, IQR 57–96; *p* = 0.003) (Table 1; Figure 3). On the other hand, the CPR quality (ET: 59%, IQR 43–75; FM: 45%, IQR 37–57; *p* = 0.001) and the ventilation quality (ET: 53%, IQR 19–86; FM: 16%, IQR 4-39; *p* < 0.001) were higher in the ET than in the FM test (Figure 3).

## 4. Discussion

During pediatric CPR, ventilation quality is a relevant outcome factor [1]. For this reason, the goal should be to reproduce what is normal for age ventilation parameters, attempting to avoid both hypo and hyperventilation, regardless of which ventilation method is used [2]. However, obtaining good ventilation quality seems to be difficult, both in real patients and with manikins [7,23]. When training, providers can rely on the CC quality feedback systems, but not enough attention has been paid to the quality of ventilations. 

In this sense, and as far as we know, our study has been the first to analyze the quality of the ventilations during CPR of an infant manikin, not only in terms of volume but also in terms of peak airway pressure generated. Our results indicate that even people with previous CPR training and with the ability to perform good quality CPR (in terms of CC) on a manikin, also have problems to achieve the ventilation targets on the manikin, especially when the technique used is the FM. If translated to real patients, this suboptimal ability could compromise the CPR result due to insufficient volumes delivered or due to excessive pressures generated in the airway with risk of barotrauma. 

We have observed that when ventilating with a FM, air entry into the airway was not achieved or insufficient air volume was achieved at a higher rate than when ventilating with ET. In addition, with this technique, a median of 53% of ventilations achieved an excessive volume and in a median of 18% of cases the pressure generated was higher than 30 cm H_2_O, indicating that less than one in four ventilations “hit the target”. During CPR courses, the emphasis is usually on achieving good airtightness between the mask and the victim’s face in order to avoid air leaks and prevent hypoventilation. However, our results alert that the opposite situation (good airtightness associated with hyperventilation) may be even more frequent. This should also be taken into account by instructors so as to encourage students to learn quality ventilation, while also emphasizing the danger of hyperventilation.

As expected, when CPR was performed with the airway isolated by an ET, all ventilations reached the lungs, and the risk of ineffective ventilations was greatly reduced. Although ventilation is significantly better with ET than with FM, and it facilitates better quality CPR, with ET the risk of ventilations with excessive volume and/or pressures greater than 30 cm H_2_O persists. The results obtained show that there is a relationship between achieving a peak pressure greater than 30 cm H_2_O and excessive ventilation volumes. This indicates that, even when the patient is intubated, the rescuer should try to be aware of the volumes breathed in and the pressures generated with each rescue breath. This problem may be quite common, as Bassani et al. reported pressure peaks greater than 40 cm H_2_O in more than half of intubated neonatal CPR simulations [24].

Priority should be given to adequate insufflation of air volume during infant resuscitation and the potential risk of volu-barotrauma if excessive pressure volume is delivered. In order to optimize ventilation, it may be a good strategy to attach a simple mechanical pressure manometer to the self-inflating bags. Therefore, this device should be recommended to be used regularly in our opinion, and not only during real patients care but also in training sessions. The visualization of the oscillations of the pressure manometer and the peak at the end of insufflation permits a quick and real time visualization of the generated pressure. This is an effective method to avoid excessive peak pressures provided an adequate chest rise of the baby is confirmed and, on the other hand, to detect partial or full airway obstruction in the event that high pressures are generated but a chest rise is not observed [1]. Previous studies have recommended its use when performing ventilations with a self-inflating bag, since at present its use is not common practice [25,26]. In any case, the rescuer must also monitor the elevation of the thorax during each insufflation (i.e., the only estimation of the insufflated volume that is available in most CPR scenarios) to ensure that the airway is clear. 

The use of a mechanical pressure manometer would also be useful during FM ventilation, where the direct monitoring of the pressure can also help prevent gastric insufflation [25] and the risk of bronchial aspiration, especially in infants. 

Two practical implications for improving ventilation in pediatric patients requiring CPR are derived from our study. Firstly, we suggest incorporating a mechanical pressure manometer into the self-inflating bag also for training. This is a very simple, useful, and inexpensive device that could also be included in the manikins for CPR training. Secondly, we encourage pediatric CPR instructors to place special emphasis on correcting effective and safe ventilations, not only to achieve good airtightness between the mask and the victim’s face, but also to avoid generating excessive pressure using a mechanical pressure manometer.

No differences were observed in the quality of chest compressions between the two ventilation techniques. However, a lower percentage of CC that were not released correctly with ET was observed. Observation by the investigators during testing suggests that these results appear to be due to ventilating and compressing at the same time. In the tests, ventilations were observed that were not fully released, made impossible by the lungs being full of air. This is likely to be a limitation of the dummy used. No evidence has been observed in the literature reflecting similar results.

Some limitations of the study have to be pointed out. On the one hand, it is an evaluation carried out under controlled conditions, with nursing students who do not have previous experience in real patient resuscitation. Furthermore, since it is a simulation study, the results cannot be directly extrapolated to clinical practice with real victims, due to the poor anatomical relationship of the airway and chest-lung of a manikin with that of a child. In this sense, this study does not aim to transfer the findings to the result of a real resuscitation, but rather to improve the skills by including a fundamental variable (pressure), which is a component that has not been contemplated so far in the training of nurses.

## 5. Conclusions

Performing quality CPR ventilations in an infant model is not an easy skill for trained nursing students, especially when the technique is with a face mask. Both with an endotracheal tube or a face mask there is a significant incidence of excessive or insufficient (in terms of volume and/or pressure) ventilations, especially in the case of the face mask. Specific training focused on the quality of ventilations delivery, guided by simple manometer devices attached to the self-inflating bag should be considered in life support training for pediatric providers. We propose that pediatric instructors consider not only CC quality but also ventilations and devote more time to this element of training, and it should be noted that excess pressure must be corrected. 

## Figures and Tables

**Table 1:** Quality of ventilations and chest compressions. Comparison of ET and FM tests.

Variable	Endotracheal Tube (ET)		Face Mask (FM)		*p*-Value
	Median	IQR	Median	IQR	
Ventilations (V)					
Frequency (breaths/min)	10	(9–13)	10	(10–11)	NS
Mean tidal volume (mL)	50	(46–56)	57	(51–69)	*p* = 0.02
Mean tidal volume (mL/kg)	9	(8–10)	10	(9–12)	*p* = 0.02
V that reached the lungs (%)	100	(100–100)	98	(81–100)	*p* < 0.001
V with insufficient volume (%)	0	(0–1)	5	(2–10)	*p* < 0.001
V with adequate volume (%)	68	(35–88)	22	(8–41)	*p* < 0.001
V with excessive volume (%)	29	(11–62)	53	(29–73)	*p* = 0.04
V with peak pressure > 30 cm H_2_O (%)	0	(0–41)	18	(0–56)	*p* = 0.03
Chest compressions (CC)					
Mean depth (mm)	40	(38–41)	41	(39–42)	NS
Mean rate (CC/min)	111	(103–119)	115	(108–119)	NS
CC with correct hand position (%)	100	(95–100)	100	(99–100)	NS
CC with adequate release (%)	81	(57–96)	93	(74–99)	*p* = 0.003

ET: Endotracheal tube; FM: Face mask; V: Ventilations; CC: Chest compressions; IQR: Interquartile range; NS: Not significant.

## Data Availability

Not applicable.
